# Species distribution, antifungal susceptibility, and clinical profiles of patients with osteoarticular fungal Infections: A retrospective study

**DOI:** 10.1016/j.nmni.2025.101676

**Published:** 2025-11-21

**Authors:** Jie Liu, Kaiming Zhang, Rui Yu, Haozhi Han, Peng Zhao

**Affiliations:** a Department of Dermatology, Shanxi Provincial People's Hospital Affiliated to Shanxi Medical University, Taiyuan, 030000, China; b Department of Orthopedic Surgery, Changsha Hospital of Traditional Chinese Medicine, 22 Xingsha Avenue, Changsha City, Hunan Province, 410100, China; c Shanxi University of Chinese Medicine Third Clinical College, China, Shanxi; d Department of Minimally Invasive Spine Surgery, Shanxi Provincial People's Hospital Affiliated to Shanxi Medical University, Taiyuan, 030000, China

**Keywords:** Osteoarticular fungal infections, Invasive fungal infections, Antifungal susceptibility, Clinical profiles, Therapy strategy

## Abstract

**Background:**

Osteoarticular fungal infections (OAFIs), including fungal osteomyelitis and septic arthritis, represent uncommon but clinically significant complications in musculoskeletal care. Current management remains challenging due to limited evidence guiding antifungal selection. This study aims to characterize the epidemiological patterns, clinical features, and antifungal susceptibility profiles of OAFIs, with particular focus on the critical role of *in vitro* susceptibility testing in determining treatment outcomes.

**Methods:**

A retrospective study of patients was conducted with OAFIs treated between January 2020 and February 2024, analyzing clinical manifestations, surgical interventions, and associated risk factors. Fungal identification was performed using matrix-assisted laser desorption/ionization time-of-flight mass spectrometry (MALDI-TOF MS), followed by broth microdilution antifungal susceptibility testing for amphotericin B (AMB), fluconazole (FLC), voriconazole (VRC), and posaconazole (POS). Minimum inhibitory concentration (MIC) values were interpreted according to CLSI guidelines to determine susceptibility profiles and identify potential resistance mechanisms.

**Results:**

Sixty fungal isolates were isolated from 60 patients with OAFIs including *Candida* spp. (n = 40, 66.7 %), *Aspergillus* spp. (n = 14, 23.3 %), *Cryptococcus neoformans* (n = 2, 3.3 %), *Trichophyton rubrum* (n = 2, 3.3 %), *Lomentospora prolificans* (n = 1, 1.7 %), and *Cryptococcus laurentii* (n = 1, 1.7 %). The isolates were obtained from joint fluid (n = 48, 80 %) and inflammatory lesions (n = 12, 20 %). Antifungal susceptibility testing demonstrated highest MIC values for FLC but susceptibility profiles for VRC and POS against all fungal isolates. Statistical analysis revealed significant differences in VRC and POS activity among *Candida*, *Aspergillus*, and *Cryptococcus* spp. (F = 15.78, *P* < 0.01; F = 66.88, *P* < 0.0001). VRC activity did not differ between *Candida* and *Aspergillus* spp., but both were lower than against *Cryptococcus* spp. (*P* < 0.05 and *P* < 0.05). POS activity was higher against *Candida* than *Aspergillus* (*P* < 0.001) and *Cryptococcus* spp. (*P* < 0.0001), and higher against *Cryptococcus* than *Aspergillus* (*P* < 0.05). Systemic comorbidities were common (73.3 %), one patient was HIV-positive, and three had only localized superficial fungal infections.

**Conclusion:**

We concluded that *Candida albicans* and *Aspergillus fumigatus* as the predominant pathogens in OAFIs, while rare species including *Cryptococcus neoformans*, *Lomentospora prolificans*, and *Cryptococcus laurentii* were also isolated from OAFI cases. Antifungal susceptibility testing revealed VRC and POS as potentially effective therapeutic options for OAFIs. These findings underscore the need for early detection of rare fungal pathogens, susceptibility-guided therapy, and continuous resistance surveillance in managing OAFIs in non-immunodeficient patients.

## 1 Introduction

Osteoarticular fungal infections (OAFIs), including fungal osteomyelitis and septic arthritis, are rare but serious infections that may lead to substantial disability, with some fungal species exhibiting opportunistic behavior in immunocompromised patients [1]. Diagnosing and treating OAFIs present considerable challenges for orthopedic surgeons due to the often subtle and gradual onset, leading to delayed recognition [2]. Recent research indicates that fungal pathogens are identified as the causative agents in approximately 0.5–1.6 % of vertebral osteomyelitis cases and account for roughly 10 % of rib osteomyelitis instances [[3], [4], [5], [6]]. Nonetheless, a consistent rise in reported cases has been observed over the past ten years [1,7]. This increasing prevalence is observable among both immunocompetent and immunocompromised patients [8,9], including protracted chemotherapy-induced neutropenia, receipt of corticosteroids, recent surgery, use of illicit intravenous drugs, use of broad-spectrum antibiotics, indwelling catheters, diabetes mellitus, HIV infection, organ transplantation, and total parenteral nutrition [9,10]. Among pathogens, *Candida* and *Aspergillus* species are most frequently associated with OAFIs due to specific characteristics like the small size of conidia facilitating tissue penetration and the presence of melanin on their surface. Current literature on OAFIs is limited to small case series and individual case reports, resulting in lower-quality evidence [11,12].

The clinical presentation of OAFIs is often nonspecific, lacking unique imaging characteristics, which complicates early detection. This may lead to misdiagnosis, delayed identification, and subsequently, a delay in appropriate treatment. Currently, the range of antifungal agents available for the treatment of OAFIs remains limited. According to the 2013 Consensus Meeting on Periprosthetic Joint Infection [13] and the 2016 Infectious Diseases Society of America (IDSA) guidelines, recommended options include amphotericin B deoxycholate, triazole antifungals, and combination therapy involving caspofungin [14,15]. The triazole antifungal agents. in particular, includes widely used fluconazole (FLC) as well as itraconazole (ITC), posaconazole (POS), and voriconazole (VRC), with POS and VRC representing second generation azoles [16]. Of particular concern is the increasing resistance of *Candida* species to frontline triazole drugs (such as FLC) and echinocandins, along with the emergence of multidrug-resistant strains, despite the limited use of echinocandins as monotherapy [17,18]. High-quality data on OAFIs treatment, especially from randomized clinical trials, systematic reviews, and meta-analyses, remain scarce, highlighting the need for further research [9]. Therefore, it is imperative to improve orthopedic physicians’ awareness of OAFIs while establishing earlier, more sensitive diagnostic protocols and therapeutic interventions.

This study aimed to characterize the epidemiological patterns, clinical manifestations, and antifungal susceptibility profiles of OAFIs, with particular emphasis on the role of *in vitro* susceptibility testing in guiding therapeutic decision. Furthermore, through comprehensive retrospective analysis, we aimed to establish evidence-based recommendations to assist clinicians in optimizing antifungal treatment regimens.

## 2 Materials and methods

### 2.1 Study design

**Fig. 1:**
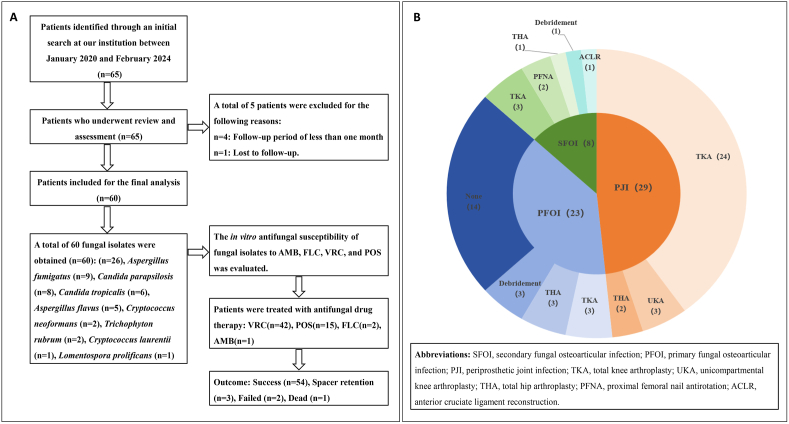
**(A)** Study flowchart. A retrospective analysis was conducted on 60 patients with OAFIs (January 2020 to February 2024). Following fungal isolation and *in vitro* susceptibility testing, patients underwent antifungal therapy. Outcomes included 54 successes, 3 cases with spacer retention, 2 failures, and 1 non-treatment-related death (heart disease). **(B)** A total of 60 patients with OAFIs, comprising 8 with secondary fungal osteoarticular infections (SFOI), 23 with primary fungal osteoarticular infections (PFOI), and 29 with periprosthetic joint infections (PJI). Surgical management following various types of osteoarticular fungal infections. The outer ring of the pie chart indicates the types of subsequent surgical procedures received, which included: total knee arthroplasty (TKA), unicompartmental knee arthroplasty (UKA), total hip arthroplasty (THA), proximal femoral nail antirotation (PFNA), anterior cruciate ligament reconstruction (ACLR), and debridement.

This study was approved by the Ethics Committee of Shanxi Provincial People's Hospital (Approval No. 2024-439). All retrospective analyses were conducted using anonymized clinical records in accordance with institutional ethical guidelines and national regulations, and the study included all cases of OAFIs diagnosed and treated at our institution between January 2020 and February 2024. We collected detailed demographic information (age, gender, side of surgery, sinus involvement, medical and surgical history), inflammatory markers, microbiology results, and antifungal susceptibility data for each patient. The study flowchart is shown in Fig. 1.

### 2.2 Inclusion and exclusion criteria

The diagnosis of OAFIs was established based on the criteria proposed by Gamaletsou et al. [19], which required the concurrent fulfillment of all three following components: (1) Relevant clinical presentation, defined by the presence of at least one of the following: persistent or worsening localized pain, swelling, or erythema despite prior antibacterial therapy; presence of a sinus tract with drainage; or recurrent fever (>38.3 °C) in the context of a recent orthopedic procedure or immunocompromised state. (2) Corresponding radiographic findings, evidenced by at least one of the following features on MRI or CT: progressive osteolysis or bone destruction; presence of a sequestrum or involucrum; or a soft-tissue abscess/phlegmon contiguous with bone. (3) Microbiological confirmation of fungi by culture from specimens obtained via open surgery or percutaneous biopsy, including bone tissue, synovial fluid, or metal hardware. Primary implant-related infections (PJI) were defined according to the Musculoskeletal Infection Society (MSIS) guidelines [13]. The study encompassed cases of primary fungal PJI, primary fungal osteomyelitis or arthritis, and fungal infections secondary to prior bacterial osteomyelitis or implant-related infections. Patients with a follow-up duration of less than one month were excluded.

### 2.3 Sample collection and processing

Joint fluid was aspirated preoperatively or during surgery. During the procedure, 3–5 samples of synovium or granulation tissue exhibiting clear inflammatory signs including hyperemia, edema, and friability, were collected. necrotic areas, identified by avascularity, pale or dark discoloration, and liquefied consistency, were carefully avoided. For fungal isolation, samples were cultured on Sabouraud Dextrose Agar (SDA, Thermo Fisher Scientific) and incubated at 28 °C for 3–7 days. The incubation period was extended to 14 days if no growth was detected initially. All original clinical samples were preserved at −20 °C in sterile containers for subsequent analysis, without the addition of any storage media.

### 2.4 Selection of antifungal agents and treatment duration

Antifungal drugs were selected based on susceptibility test outcomes after consultation with infectious disease specialists. In the absence of systemic infection, antibiotics were not administered prior to sampling. Upon confirmation of fungal infection, specific antifungal therapy was initiated, generally consisting of intravenous antifungal treatment followed by oral medication for a total of six months, as recommended by the 2013 Consensus Meeting on Periprosthetic Joint Infection [13] and 2016 IDSA guidelines [15]. In brief, the FLC, AMB, POS, and VRC were included based on their recommended roles in the management of invasive fungal infections as the referenced IDSA guidelines [15]. Specifically, FLC and echinocandins are first-line agents for initial and step-down therapy, while echinocandins were used as part of combination therapy but not used as monotherapy [18,20]. VRC and POS are broader-spectrum triazoles with activity against various fungi. It is important to note that *Aspergillus* spp. are intrinsically resistant to FLC; therefore, FLC susceptibility was not listed for *Aspergillus* spp. The duration and sequence of therapy were aligned with guideline recommendations, emphasizing a total treatment duration of up to six months for deep-seated infections such as periprosthetic joint infections, with adjustments based on clinical response and susceptibility results.

For patients undergoing two-stage revision arthroplasty, a minimum of six months of oral antifungal therapy was administered based on susceptibility results prior to reimplantation. For single-stage arthroplasty, the new total hip or knee prosthesis was implanted. For two-stage revision, an antibiotic-impregnated cement spacer was inserted. Normally, AMB (200 mg) or VRC (300 mg) was added per 40 g of bone cement to prepare the spacer [20,21]. After surgery, patients were prescribed with systemic antifungal treatment for at least 3 months.

### 2.5 Isolation and identification of fungal species

This retrospective, laboratory-based study analyzed fungal infection cases recorded between January 2020 and February 2024. Only one unique fungal isolate per patient was included. Data on specimen collection dates, types, and anatomical sites were systematically documented. All specimens underwent direct microscopic examination with potassium hydroxide (KOH) to facilitate rapid preliminary detection of fungal elements. Initial identification was based on morphological characteristics at a central laboratory. For definitive species-level identification, isolates were subjected to matrix-assisted laser desorption/ionization time-of-flight mass spectrometry (MALDI-TOF MS) using the Bruker Biotyper® system (Bruker Daltonics). Measurements were carried out on a Microflex LT/SH instrument, and resulting spectra were analyzed against the MBT Compass Library (version 11.0). Consistent with established criteria, isolates with log(score) values ≥ 2.0 were considered confidently identified at the species level [22,23].

### 2.6 Antifungal susceptibility testing

Antifungal susceptibility testing was conducted in accordance with Clinical and Laboratory Standards Institute (CLSI) guidelines (M27-A3, S4, M38-A2, and M59) [[24], [25], [26], [27]]. In brief, the broth microdilution method was employed to determine the susceptibility of isolates to FLC (Sigma-Aldrich, USA), VRC (Sigma-Aldrich, USA), AMB (Sigma-Aldrich, USA), and POS (Sigma-Aldrich, USA). Fungal conidia were enumerated using a Neubauer chamber and adjusted to a final concentration of 1 × 10^6^ CFU/mL [[24], [25], [26], [27]]. Serial two-fold dilutions of each antifungal agent were prepared in 96-well microplates across a concentration range of 0.03–64 μg/mL. The inoculated plates were incubated at 35 °C according to CLSI guidelines, with incubation times of 24 h for *Candida* spp., 72 h for *Cryptococcus* spp., 48 h for *Aspergillus* spp., 72 h for *Lomentospora prolificans*, and 4–5 days for *Trichophyton rubrum*. Minimum inhibitory concentration (MIC) values were interpreted according to relevant CLSI documents: M38-A2 for molds and M27-A3/S4 for yeasts. The MIC for AMB against all fungi was defined as the lowest concentration resulting in 100 % growth inhibition. For azole agents against yeasts, the MIC corresponded to a 50 % reduction in growth, whereas for molds, it reflected complete (100 %) growth inhibition. Quality control was ensured by testing reference strains *Aspergillus flavus* ATCC® 204304 and *Candida parapsilosis* ATCC® 22019, with observed MICs verified against established reference ranges. All experiments were performed in duplicate.

### 2.7 Outcome measurement

Treatment success was defined by the following criteria [13]: (1) absence of infection-related symptoms, such as pain, swelling, sinus tract, or local warmth, with well-healed incisions; (2) normalization of inflammatory markers, including white blood cell count, C-reactive protein (CRP), and erythrocyte sedimentation rate (ESR); and (3) no radiological evidence of infection, including bone resorption or osteolysis. Upon confirmation of infection eradication, patients underwent second-stage revision surgery, which involved removal of the spacer, implantation of a new prosthesis, and placement of a drain, which was typically removed within 48 h.

Treatment failure was defined as infection relapse, the need for additional surgical intervention, or the requirement for lifelong antifungal suppression. Cases in which antifungal-impregnated cement spacers were permanently retained due to patient ineligibility for, or refusal of, second-stage reimplantation were categorized separately as spacer retention.

### 2.8 Statistical analysis

Statistical analysis was performed using SPSS software (version 18.0; SPSS Inc., Chicago, IL, USA). Data in the tables are expressed as case numbers (n) and percentages (%). For comparisons among three groups of quantitative data, one-way analysis of variance (ANOVA) was applied when the data satisfied the assumptions of normality and homogeneity of variance; otherwise, the Kruskal–Wallis test was used. Post hoc pairwise comparisons were conducted with Bonferroni correction where appropriate. Comparisons between two groups were performed using Student's t-test. Descriptive statistics were used to summarize the data, with quantitative variables presented as means ± standard deviations (SD) and categorical variables presented as frequencies and percentages. A two-tailed *P* < 0.05 was considered statistically significant.

## 3 Results

### 3.1 Study population and clinical profile of patients

A total of 65 patients were identified from the registry. Four were excluded due to a follow-up period of less than one month, one was lost to follow-up, leaving 60 patients for analysis. The study flowchart is shown in Fig. 1. Fungal pathogens were isolated from all 60 patients, confirming the diagnosis of fungal arthritis. The male-to-female ratio was 29 (48 %) to 31 (52 %). According to the age of the included patients, 3 (5 %) of the patients were less than 40 years old, 26 (43 %) of the patients were between 40 and 60 years old, and 31 (52 %) of the patients were over 60 years old. The laboratory assessment primarily involved the microbial culture of synovial fluid or tissue specimens. The average serum levels of CRP and ESR were found to be 21.0 ± 9.0 mg/L (with a range of 5.9–39.6) and 36.9 ± 35.3 mm/h (ranging from 1.0 to 117.0), respectively. Additionally, the mean synovial WBC count and the percentage of polymorphonuclear (PMN) cells were (6731 ± 2169) × 10^6^/L (with a range of 2650–15,070 × 10^6^/L) and 60.3 % ± 12.3 % (ranging from 40.2 % to 80.0 %), respectively.

Among the 60 patients, 29 had periprosthetic joint infection (PJI), including 24 after total knee arthroplasty (TKA), 2 after total hip arthroplasty (THA), and 3 after unicompartmental knee arthroplasty (UKA). Primary fungal osteoarticular infection (PFOI) was observed in 23 patients, including 3 after TKA, 3 after THA, 3 following debridement, and 14 without prior surgery. The remaining 8 patients had secondary fungal osteoarticular infection (SFOI), including 3 after TKA, 1 after THA, 1 after anterior cruciate ligament (ACL) reconstruction, 2 after proximal femoral nail antirotation (PFNA) procedures, and 1 after debridement. Demographic and clinical information of patients was represented in Fig. 1. Among the 60 patients, 47 (78.3 %) had at least one comorbid condition. Of these, 44 patients had systemic comorbidities, including coronary heart disease (CHD), hypertension (HT), diabetes mellitus (DM), renal impairment, hepatitis B (HBV), hypoalbuminemia, HIV infection (1 patient), chronic pneumonia, hyperthyroidism, anemia, or uremia. The remaining three patients (Cases 19, 25, and 41) had only localized superficial fungal infections.

### 3.2 Fungal species distribution and antifungal susceptibility test results

A total of 60 unique fungal isolates were obtained, comprising 40 *Candida* spp., 14 *Aspergillus* spp., 2 *Cryptococcus neoformans*, 2 *Trichophyton rubrum*, 1 *Lomentospora prolificans*, and 1 *Cryptococcus laurentii*. Interestingly, concomitant cutaneous mycoses were reported in case 25, case 41, and case 59. Among the two patients from whom *Cryptococcus neoformans* was isolated, case 15 was also diagnosed with HIV, whereas case 30 had received a renal transplant five years earlier. In 60 patients OAFIs was diagnosed was diagnosed by positive cultures based on morphological features. Notably, MALDI-TOF MS systems were successfully implemented for accurate fungal species identification throughout this investigation, demonstrating their reliability in mycological diagnostics. The specimens primarily consisted of joint fluid (48 samples) and inflammatory lesion tissues (12 samples). Consequently, 12 fungal species were identified through both culture and histological examination (see Table 1).

**Table 1:** Clinical characteristics of 60 patients with fungal osteoarticular infection (FOI).

Fungal species	Sex (M/F)	Age	Underlying condition	SF WBC (x10^6^/L)	PMN (%)	CRP (mg/L)	ESR (mm/h)	Treatment	FU (months)	Outcome
*Candida albicans* (26)	15/11	61.3 ± 10.2	None (7), HT (12), DM (4), CHD (1), Pneumonia (1), Renal dysfunction(1)	6930 ± 2534	63.3 ± 12.9	20.0 ± 8.0	31.9 ± 34.6	TR + POS (12), TR + VRC (9) TR + AMB (1), TR + FLC (1), Arthroscopic debridement + POS (1), THA + VRC (1), Long-term VRC (1)	25.0 ± 10.4	Success (26)
*Candida parapsilosis* (8)	4/4	64.0 ± 10.1	None (4), HT (1), DM (1), HT + DM (1), Uremia + DM + HT + hypoalbuminemia, anemia (1)	6175 ± 2460	65.5 ± 11.3	21.3 ± 11.6	35.0 ± 34.5	TR + VRC (6), PFNA + FLC (1), TKA + VRC (1)	21.3 ± 10.2	Success (7) Failed (1)
*Candida tropicalis* (6)	3/3	65.8 ± 15.5	DM (1), HT + DM (1),Hyperthyroidism (2), Tinea pedis (1)	7019 ± 2652	54.2 ± 7.6	23.7 ± 11.6	55.5 ± 33.0	TR + VRC (3), TKA + POS (1) TKA + VRC (2)	18.0 ± 5.7	Success (3) Spacer retention (1) Failed (1) Dead (1)
*Aspergillus fumigatus* (9)	4/5	55.8 ± 19.0	None (1), HT (2), DM (2), CHD (1), HT + DM (1), DM + hypoalbuminemia (1), HT + chronic pneumonia + liver cyst (1)	6491 ± 1349	51.0 ± 8.5	25.7 ± 8.2	50.1 ± 49.3	Arthroscopic debridement + VRC (1), TR + VRC (4), TR + POS (1), Long-term VRC (2), OR + VRC (1)	18.9 ± 8.9	Success (7) Spacer retention (2)
*Aspergillus flavus* (5)	2/3	58.8 ± 6.1	HT (2), CHD (1), DM + HBV(1), Renal dysfunction (1)	6968 ± 1326	59.0 ± 16.0	21.1 ± 7.4	33.2 ± 30.2	TR + VRC (5)	32.0 ± 12.5	Success (5)
*Cryptococcus neoformans* (2)	1/1	68.5 ± 14.8	HIV (1), CHD + Renal + transplantation (1)	5621 ± 745	57.7 ± 16.3	19.3 ± 5.2	27.0 ± 17.0	Arthroscopic debridement + VRC (1), TR + VRC (1)	18.5 ± 22.0	Success (2)
*Cryptococcus laurentii* (1)	0/1	82	HT + DM (1)	4500	63.3	11.3	52.0	Debridement + fusion + VRC (1)	40.0	Success (1)
*Trichophyton rubrum* (2)	0/2	59.5 ± 3.5	None (1), Tinea pedis (1)	7130 ± 320	65.1 ± 8.2	16.6 ± 9.8	18.0 ± 1.4	TR + VRC (2)	28.0 ± 00.0	Success (2)
*Lomentospora prolificans* (1)	0/1	68	Renal dysfunction (1)	8875	58.1	6.5	18.0	TR + VRC (1)	31.0	Success (1)
Total	29/31	61.7 ± 11.4	None (13), CHD (4), DM (7), DM + bilateral kidney stone + HBV (1), DM + hypoalbuminemia(1), DM + HBV(1), HIV(1), HT(17), HT + chronic pneumonia + liver cyst(1), HT + DM(4), Hyperthyroidism (2), Pneumonia(1), Renal dysfunction (3), Tinea pedis(3), Renal dysfunction (3), Uremia + DM + HT + hypoalbuminemia + anemia (1)	6731 ± 2169	60.3 ± 12.3	21.0 ± 8.9	37.0 ± 35.2	Arthroscopic debridement + POS (1), Arthroscopic debridement + VRC (2), Debridement + fusion + VRC(1) Long-term VRC (3), OR + VRC (1), PFNA + FLC (1), THA + VRC (1), TKA + POS (1), TKA + VRC (3), TR + POS (13), TR + VRC (31), TR + AMB (1), TR + FLC (1)	41.4 ± 10.6	Success (54) Spacer retention (3) Failed (2) Dead (1)

**Abbreviations:** HT, hypertension; DM, diabetes; HIV, human immunodeficiency virus; HBV, hepatitis B; OA, osteoarthritis; TB, tuberculosis; RA, rheumatoid arthritis; ONFH, osteonecrosis of the femoral head; TKA, total knee arthroplasty; UKA, unicompartmental knee arthroplasty; THA, total hip arthroplasty; PFNA, proximal femoral nail antirotation; ACLR, anterior cruciate ligament reconstruction; DAIR, debridement, antibiotics, and implant retention; abx, antibiotics; SF, synovial fluid; WBC, white blood cell; PMN, polymorphonuclear leukocyte; FU, follow-up; OR, one-stage revision; TR, two-stage revision; FLC, fluconazole, VRC, voriconazole; AMB, amphotericin B; POS, posaconazole. Spacer retention, antifungal-impregnated cement spacer retention.

MIC_50_ and MIC_90_ values were not reported for fungal groups comprising fewer than five isolates due to the limited statistical reliability associated with small sample sizes. Among *Candida albicans* (26 strains), the MIC_50_ and MIC_90_ to FLC were 16 μg/mL and 64 μg/mL, respectively, with a geometric mean (GM) MIC of 3.232 μg/mL. POS exhibited a MIC range of 0.03–0.25 μg/mL and a GM of 0.094 μg/mL. For *Candida parapsilosis* (8 strains), the MIC_50_ and MIC_90_ of FLC were 0.25 μg/mL and 0.5 μg/mL, respectively (GM: 0.273 μg/mL), while VRC showed a MIC range of 0.03–0.125 μg/mL (GM: 0.047 μg/mL). In the case of *Candida tropicalis* (6 strains), the MIC_50_ and MIC_90_ of FLC were 16 μg/mL and 32 μg/mL (GM: 22.627 μg/mL). POS and VRC against this species showed GM values of 0.156 μg/mL (range: 0.03–0.5 μg/mL) and 0.110 μg/mL (range: 0.06–0.25 μg/mL), respectively. For *Aspergillus fumigatus* (9 strains), VRC showed a MIC range of 0.06–0.25 μg/mL (GM: 0.083 μg/mL), and AMB exhibited a range of 0.5–2 μg/mL (GM: 1.167 μg/mL). Similarly, for *Aspergillus flavus* (5 strains), the MIC range for VRC was 0.125–0.25 μg/mL (GM: 0.165 μg/mL), while that for AMB was 0.5–2 μg/mL (GM: 1.000 μg/mL). Other tested species included *Cryptococcus neoformans*, *Cryptococcus laurentii*, *Lomentospora prolificans*, and *Trichophyton rubrum*. These isolates generally exhibited the highest MICs to FLC and the lowest to VRC. Notably, *Trichophyton rubrum* was isolated from two patients (Cases 41 and 59), both of whom presented with cutaneous mycotic infections. Statistical analysis revealed significant differences in VRC and POS activity among *Candida*, *Aspergillus*, and *Cryptococcus* spp. (F = 15.78, *P* < 0.01; F = 66.88, *P* < 0.0001). VRC activity did not differ between *Candida* and *Aspergillus* spp., but both were lower than against *Cryptococcus* spp. (*P* < 0.05 and *P* < 0.05). POS activity was higher against *Candida* than *Aspergillus* (*P* < 0.001) and *Cryptococcus* spp. (*P* < 0.0001), and higher against *Cryptococcus* than *Aspergillus* (*P* < 0.05) (see Table 2).

**Table 2:** *In vitro* antifungal susceptibility profiles of fungal isolates. MIC values are presented descriptively as range and median for groups with n ≥ 3 isolates, and as range only for n = 1–2. The MIC_50_, MIC_90_, and GM were not calculated for groups with fewer than 5 isolates due to limited statistical reliability.

Fungal species	Number of Strains	Antifungal agents	MIC (μg/mL)					Antifungal susceptibility (n)		
			Range	50 %	90 %	GM	Median MIC			
*Candida albicans*	26	FLC	0.125->64	16	64	3.232	19.197	S (12)	I (0)	R (14)
		VRC	0.06–0.500	0.125	0.5	0.137	0.182	S (18)	I (8)	R (0)
		POS	0.03–0.250	0.125	0.25	0.094	0.127	S (18)	I (8)	R (0)
		AMB	0.125–1	0.5	1	0.404	0.563	WT (26)	NWT(0)	
*Aspergillus fumigatus*	9	VRC	0.06–0.25	0.06	0.25	0.083	0.096	WT (9)	NWT(0)	
		POS	0.125–2	0.5	1	0.429	0.611	WT (7)	NWT(2)	
		AMB	0.5–2	1	2	1.167	1.278	WT (6)	NWT(3)	
*Candida parapsilosis*	8	FLC	0.125–0.5	0.25	0.5	0.273	0.328	S (8)	I (0)	R (0)
		VRC	0.03–0.125	0.03	0.125	0.047	0.058	S (8)	I (0)	R (0)
		POS	0.03–0.5	0.125	0.5	0.135	0.211	S (5)	I (3)	R (0)
		AMB	0.06–0.25	0.125	0.25	0.148	0.164	WT (8)	NWT(0)	
*Candida tropicalis*	6	FLC	16–32	16	32	22.627	24.000	S (0)	I (0)	R (6)
		VRC	0.06–0.25	0.125	0.25	0.110	0.124	S (5)	I (1)	R (0)
		POS	0.03–0.5	0.25	0.5	0.156	0.213	S (4)	I (2)	R (0)
		AMB	0.25–1	0.5	1	0.561	0.667	WT (6)	NWT(0)	
*Aspergillus flavus*	5	VRC	0.125–0.25	0.125	0.25	0.165	0.175	WT (5)	NWT(0)	
		POS	0.125->4	0.5	4	0.574	1.175	WT (5)	NWT(0)	
		AMB	0.5–2	1	2	1.000	1.100	WT (4)	NWT(1)	
*Cryptococcus neoformans*	2	FLC	1–2	NA			1.500	WT (2)	NWT(0)	
		VRC	0.03–0.06	NA			0.045	WT (2)	NWT(0)	
		POS	0.25–0.5	NA			0.375	WT (1)	NWT(1)	
		AMB	0.25–1	NA			0.625	WT (1)	NWT(1)	
*Trichophyton rubrum*	2	FLC	2–4	NA			3.000	NA		
		VRC	0.03–0.06	NA			0.045	NA		
		POS	0.25–0.5	NA			0.375	NA		
		AMB	0.25–1	NA			0.625	NA		
*Cryptococcus laurentii*	1	FLC	MIC = 2 μg/mL				2.000	WT (1)	NWT(0)	
		VRC	MIC = 0.06 μg/mL				0.060	WT (1)	NWT(0)	
		POS	MIC = 0.25 μg/mL				0.250	WT (1)	NWT(0)	
		AMB	MIC = 0.25 μg/mL				0.250	WT (1)	NWT(0)	
*Lomentospora prolificans*	1	FLC	MIC = 4 μg/mL				4.000	NA		
		VRC	MIC = 0.125 μg/mL				0.125	NA		
		POS	MIC = 0.25 μg/mL				0.250	NA		
		AMB	MIC = 0.5 μg/mL				0.500	NA		

**Abbreviations:** FLC, fluconazole, VRC, voriconazole; AMB, amphotericin B; POS, posaconazole; GM, geometric mean; MIC, minimum inhibitory concentration; S, susceptible; R, resistant; 50 and 90 %, MIC encompassing 50 and 90 % of isolates tested, respectively; NA, not applicable; WT, wild-type; NWT, non-wild-type.

### 3.3 Treatment and outcome

The overall treatment success rate was 95 % (57/60) in patients who received antifungal agents to which the causative pathogen was susceptible. Treatment success was rigorously defined as the resolution of local symptoms, normalization of inflammatory markers, absence of radiographic evidence of infection (including prosthesis loosening, bone resorption, or osteolysis) at the final follow-up, and surgical confirmation of infection eradication. Over a median follow-up of 41.4 months (range: 19–62 months), no recurrent infections requiring further surgical intervention were documented. None of the successfully treated patients required lifelong antifungal therapy, and all tolerated prolonged antifungal treatment well, with no significant adverse effects reported. Additionally, three patients (Patients 6, 7, and 8) declined second-stage reimplantation and were successfully managed with spacer retention. Treatment failure, characterized by relapse and the need for additional surgical intervention, occurred in two patients. Both patients (Case 5 infected with *Candida parapsilosis* and Case 35 infected with *Candida tropicalis*) achieved clinical success only with POS after prior VRC therapy had failed, despite their isolates exhibiting identical *in vitro* MICs to VRC and POS. One patient (Patient 21, 87 years old) died from acute myocardial infarction six months after initial antifungal surgery. The death was assessed as unrelated to the fungal infection or its treatment (VRC) and was attributed to pre-existing hypertension and diabetes.

## 4 Discussion

Osteoarticular fungal infections (OAFIs), though rare, remain clinically significant due to their diagnostic difficulty and the high risk of delayed treatment or misdiagnosis, which in severe cases may increase mortality. The limitations of conventional diagnostic approaches, including the low specificity of imaging and the limited sensitivity of cultures, further hinder timely recognition [28]. Although non-surgical management is generally preferred for spinal fungal infections, treatment outcomes are frequently compromised by antifungal resistance and relapse. The lack of prospective studies or meta-analyses continues to restrict evidence-based guidance, while current systemic fungal infection guidelines provide insufficient specificity for OAFIs, particularly given the heterogeneous susceptibility patterns of the pathogens involved [29].

In our study, *Candida* and *Aspergillus* spp. were the predominant causative fungi, consistent with previous reports of peri-prosthetic and post-surgical infections [30]. Among *Candida* isolates, *Candida albicans* and *Candida parapsilosis* were most common, while *Aspergillus fumigatus* and *Aspergillus flavus* predominated among molds. Species identification was performed using morphological methods and MALDI-TOF MS [31,32], although the absence of CHROMagar™ confirmation for *Candida* represents a methodological limitation. Beyond these established pathogens, we identified several less common fungi of notable epidemiological concern. These included *Lomentospora prolificans*, *Cryptococcus laurentii*, and invasive *Trichophyton rubrum*, each of which highlights emerging or re-emerging patterns in the etiological spectrum of OAFIs. Of particular concern was the isolation of *Lomentospora prolificans* from a 68-year-old female with secondary OAFI, given its recognized intrinsic multidrug resistance and high virulence. Although the present isolate showed susceptibility to VRC and POS, this contrasts with the well-documented resistance profile [33,34], underscoring the importance of individualized antifungal susceptibility testing. First described in 1984 [35], *Lomentospora prolificans* rarely infects immunocompetent individuals and typically necessitates a combination of surgical intervention and antifungal therapy, highlighting the importance of ongoing susceptibility surveillance. Supporting evidence includes cases such as invasive sinusitis with elevated MICs (AMB: 2, FLC: >64, VRC: >8 μg/mL) ^35^ and a life-threatening elbow infection in a pediatric patient that was managed surgically with adjunctive local and systemic antifungals [36]. Similarly, Johnston et al. reported a limb-threatening *Lomentospora prolificans* elbow infection in a 3-year-old immunocompetent boy following a closed fracture, which was successfully treated with a multimodal regimen comprising combined antifungal therapy, voriconazole-impregnated bone cement, and antiseptic joint irrigation [37]. *Cryptococcus laurentii*, historically regarded as a saprophytic organism [38,39], was isolated from a patient with renal dysfunction. Antifungal susceptibility testing revealed greater sensitivity to VRC and POS compared to FLC. While extensive antifungal susceptibility data exist for *Cryptococcus neoformans* and *Cryptococcus gattii*, studies on *Cryptococcus laurentii* remain limited [40,41]. In according with established criteria, most *Cryptococcus laurentii* isolates exhibit higher susceptibility to VRC, AMB than to itraconazole and FLC. Consistent with this pattern, our isolate demonstrated enhanced sensitivity to VRC and POS relative to FLC, underscoring the importance of patient-specific susceptibility profiling. The invasive potential of *Trichophyton rubrum*, a dermatophyte predominantly associated with superficial mycoses, is increasingly being documented. This is evidenced not only by rare infections in immunocompromised hosts, such as the post-transplant case reported by Akay et al. but also by our two cases of periprosthetic knee infection [42]. Our findings, which implicate both hematogenous spread and direct inoculation as possible routes, indicate that deep tissue invasion can occur even in the absence of significant immunosuppression. Together, these observations firmly establish *Trichophyton rubrum* as an emerging cause of invasive fungal infection that merits increased clinical attention.

Clinical study revealed important host-related risk factors for OAFIs. Azzam et al. reported that in *Candida*-related prosthetic joint infections, 68.5 % of patients had systemic comorbidities, 24.6 % were immunocompromised, and 5.4 % had a history of prolonged antibiotic use [43]. In our study, 78.3 % of patients with OAFIs had systemic comorbidities such as diabetes, hypertension, HIV infection, or renal dysfunction. Notably, several patients had concurrent superficial fungal infections (such as tinea pedis and tinea cruris), suggesting that these may serve as reservoirs for invasive disease following surgery or prosthesis placement. This raises the possibility that preoperative screening and treatment of superficial mycoses in arthroplasty candidates could represent a valuable preventive measure, though this strategy remains clinically unexplored. In Patient 41, *Trichophyton rubrum* was isolated from both the osteoarticular infection and the coexisting tinea pedis lesion, with the species identification confirmed through cultural morphology and MALDI-TOF MS analysis. Patient 25 had tinea pedis due to *Candida albicans*, while the osteoarticular infection involved *Candida tropicalis*. Patient 59 presented with concurrent osteoarticular infection and tinea cruris, both caused by *Trichophyton rubrum*. These patterns, observed in patients with prolonged antibiotic exposure, consistently involved pathogens such as *Candida albicans* and *Trichophyton rubrum*. This aligns with the systematic analysis by Cho et al. [44] of 520 knee osteoarthritis patients, which associated reduced mobility and impaired self-care with increased fungal infection risk, further underscoring the multifactorial etiology of OAFIs.

Osteoarticular fungal infections present considerable diagnostic and therapeutic difficulties, frequently resulting in delayed recognition and suboptimal clinical outcomes [43,45]. While echinocandins act as first-line treatment for invasive candidiasis through noncompetitive inhibition of (1,3)-β-D-glucan synthase, the IDSA 2016 guidelines recommend FLC either as primary monotherapy or as consolidation following short-course echinocandin induction for *Candida* osteoarticular infections (Recommendation #XI) [13,15]. Reflecting real-world practice, Gamaletsou et al. and Zhang et al. reported predominant use of AMB, FLC, or VRC, with echinocandins generally reserved for combination therapy rather than monotherapy [18,20]. Accordingly, our focus on azoles and AMB aligns with the predominant antifungal strategies during the study period. Although azoles constitute first-line therapy for systemic mycoses with FLC being widely used owing to its favorable bioavailability and safety profile that their efficacy in deep musculoskeletal infections can be limited [46]. Current IDSA guidelines specifically recommend itraconazole (200–400 mg/day) or AMB (0.7–1.0 mg/kg/day) as preferred treatments for osteoarticular mycoses [47]. These recommendations are supported by strong clinical evidence, including a meta-analysis reporting 97 % treatment success in osteoarticular blastomycosis with monotherapy using either agent [2,47]. In our study, *in vitro* susceptibility testing indicated that 73.3 % of isolates were sensitive to VRC. One patient (Patient 21) died from acute myocardial infarction six months after initial antifungal surgery; this mortality was deemed unrelated to fungal infection or its treatment (VRC), but rather associated with pre-existing hypertension and diabetes at advanced age. However, clinical failure occurred in two cases (Cases 5 and 35) despite documented *in vitro* susceptibility. This discordance underscores a recognized limitation of current antifungal susceptibility testing [48].

A representative example involved two patients (Cases 5 and 35) infected with *Candida parapsilosis* and *Candida tropicalis*, respectively. Although both isolates showed identical *in vitro* MICs to VRC and POS, the patients only achieved clinical success with POS after VRC failed. This discrepancy may be explained by pharmacokinetic and pharmacodynamic differences between the two azoles [49]. While both inhibit ergosterol synthesis, POS exhibits a broader spectrum, distinct tissue penetration, and a potentially more favorable drug-interaction profile, despite its higher acquisition cost. Although *in vitro* data often suggest superior activity of VRC against these *Candida* species [2,50,51], accumulating clinical evidence supports the efficacy of POS in azole-refractory candidiasis [52,53]. Our findings thus reinforce the view that POS represents a valuable alternative, particularly when first-line azoles fail or are poorly tolerated, highlighting the need to integrate susceptibility data with clinical judgment. AMB was administered systemically in three *Candida* osteomyelitis cases (Patients 55, *Candida albicans*), resulting in complete resolution. Notably, VRC and POS displayed the low MIC values *in vitro*, suggesting superior pharmacodynamic activity. Nevertheless, the observed clinical cure rate for POS was lower than that for VRC, again underscoring a disconnect between *in vitro* results and therapeutic performance. Management of fungal osteomyelitis remains challenging, with conflicting outcomes reported in the literature. Arranz-Caso et al. [54] described a case of zygomatic candidal osteomyelitis in a diabetic patient that resolved completely with AMB after FLC failure. In contrast, Kim et al. [55] observed clinical resistance to AMB in deep fungal infections, possibly reflecting variations in pathogen susceptibility, infection site, or host immunity. Finally, although FLC is not recommended for mold infections and is excluded from standardized mold panels (CLSI M38, EUCAST), we included it in our initial screening. Interestingly, three *Aspergillus* isolates (two *Aspergillus fumigatus* and one *Aspergillus flavus*) exhibited lower MICs (32 μg/mL) compared to other isolates (>64 μg/mL). While clinically irrelevant, this pattern aligns with sporadic experimental reports [56] and may warrant further mechanistic study.

The broader implications of our findings extend to infection control and public health preparedness. The isolation of environmentally ubiquitous fungi such as *Lomentospora prolificans* emphasizes the importance of strict intraoperative aseptic protocols and optimized air filtration systems to minimize exogenous inoculation risk. The invasive potential of dermatophytes raises the possibility that superficial fungal infections could serve as overlooked reservoirs for invasive disease, suggesting a role for targeted preoperative screening in selected patients. Finally, the intrinsic resistance patterns of emerging pathogens highlight the need for antifungal stewardship and expanded regional laboratory capacity for accurate identification and susceptibility testing. Together, these measures are essential for guiding effective therapy and enhancing preparedness for resistant fungal threats.

Several limitations should be acknowledged. Identification relied primarily on MALDI-TOF MS without routine echinocandin susceptibility testing, limiting comprehensive resistance profiling. Although echinocandins are not recommended as monotherapy for OAFIs, future prospective studies should incorporate them to further clarify their role. In addition, the relatively modest sample size of 60 patients reflects the rarity of OAFIs and reduces the statistical power to detect significant associations, thereby limiting external validity. Larger, multicenter studies are needed to confirm these observations and further define evolving epidemiological patterns.

## 5 Conclusion

OAFIs, while uncommon, pose substantial clinical challenges due to their diagnostic complexity and formidable treatment requirements, particularly in patients with underlying comorbidities, extensive surgical histories, or prolonged antibiotic exposure. Achieving favorable outcomes depends on early suspicion and heightened clinical vigilance, especially given the constraints of conventional microbiological diagnostics. Although the advent of advanced identification techniques and standardized antifungal susceptibility testing has refined management, persistent discrepancies between *in vitro* susceptibility and *in vivo* efficacy underscore a critical knowledge gap. Successful treatment generally necessitates a multidisciplinary strategy, integrating timely surgical intervention with prolonged, targeted antifungal therapy. Future efforts should prioritize large-scale prospective studies to establish clinically relevant breakpoints for principal pathogens, decipher the molecular mechanisms driving therapeutic failures despite apparent drug susceptibility, and expand AFST profiles to include cornerstone agents like echinocandins. Such initiatives are indispensable for optimizing management protocols and ultimately improving prognoses in this demanding infection domain.

## CRediT authorship contribution statement

**Jie Liu:** Writing – original draft, Validation. **Kaiming Zhang:** Software, Data curation. **Rui Yu:** Software, Resources. **Haozhi Han:** Software, Resources, Methodology. **Peng Zhao:** Writing – review & editing, Supervision.

## Ethics approval and consent to participate

The study was conducted in accordance with the principles of the Declaration of Helsinki, and the study protocol was reviewed and approved by the Ethics Committee of Shanxi Provincial People's Hospital (Approval No. 2024-439).

## Data availability statement

The data presented in this study are available on request from the corresponding authors.

## Declaration of generative AI and AI-assisted technologies

We declare that no generative AI or AI-assisted technologies were used in the writing or preparation of this work.

## Funding

This work was supported by the Basic Research Program of Shanxi Province (No. 202403021212208).

## Declaration of competing interest

The authors declare that they have no known competing financial interests or personal relationships that could have appeared to influence the work reported in this paper.

## References

[1] Papachristou S.G., Iosifidis E., Sipsas N.V., Gamaletsou M.N., Walsh T.J., Roilides E. (2020). Management of osteoarticular fungal infections in the setting of immunodeficiency. Expert Rev Anti Infect Ther.

[2] Gamaletsou M.N., Rammaert B., Brause B., Bueno M.A., Dadwal S.S., Henry M.W. (2022). Osteoarticular mycoses. Clin Microbiol Rev.

[3] Corr P.D. (2011). Musculoskeletal fungal infections. Semin Muscoskel Radiol.

[4] Cottle L., Riordan T. (2008). Infectious spondylodiscitis. J Infect.

[5] Gouliouris T., Aliyu S.H., Brown N.M. (2010). Spondylodiscitis: update on diagnosis and management. J Antimicrob Chemother.

[6] Bishara J., Gartman-Israel D., Weinberger M., Maimon S., Tamir G., Pitlik S. (2000). Osteomyelitis of the ribs in the antibiotic era. Scand J Infect Dis.

[7] Henry M., Miller A.O., Walsh T.J., Brause B.D. (2017). Fungal musculoskeletal infections. Infect Dis Clin.

[8] Arias F., Mata-Essayag S., Landaeta M.E., Capriles C.H., Perez C., Nunez M.J. (2004). Candida albicans osteomyelitis: case report and literature review. Int J Infect Dis.

[9] Bariteau J.T., Waryasz G.R., McDonnell M., Fischer S.A., Hayda R.A., Born C.T. (2014). Fungal osteomyelitis and septic arthritis. J Am Acad Orthop Surg.

[10] Smith J.W., Piercy E.A. (1995). Infectious arthritis. Clin Infect Dis.

[11] Koutserimpas C., Chamakioti I., Raptis K., Alpantaki K., Vrioni G., Samonis G. (2022). Osseous infections caused by aspergillus species. Diagnostics.

[12] Gabrielli E., Fothergill A.W., Brescini L., Sutton D.A., Marchionni E., Orsetti E. (2014). Osteomyelitis caused by Aspergillus species: a review of 310 reported cases. Clin Microbiol Infect.

[13] Parvizi J., Gehrke T., Chen A.F. (2013). Proceedings of the international consensus on periprosthetic joint infection. Bone Joint Lett J.

[14] Wang T., Xiu J., Zhang Y., Wu J., Ma X., Wang Y. (2017). Transcriptional responses of Candida albicans to antimicrobial peptide MAF-1A. Front Microbiol.

[15] Pappas P.G., Kauffman C.A., Andes D.R., Clancy C.J., Marr K.A., Ostrosky-Zeichner L. (2016). Clinical practice guideline for the management of candidiasis: 2016 update by the infectious diseases society of America. Clin Infect Dis.

[16] Donovan F.M., Shubitz L., Powell D., Orbach M., Frelinger J., Galgiani J.N. (2019). Early events in Coccidioidomycosis. Clin Microbiol Rev.

[17] Vitenshtein A., Charpak-Amikam Y., Yamin R., Bauman Y., Isaacson B., Stein N. (2016). NK cell recognition of Candida glabrata through binding of NKp46 and NCR1 to fungal ligands Epa1, Epa6, and Epa7. Cell Host Microbe.

[18] Gamaletsou M.N., Rammaert B., Bueno M.A., Sipsas N.V., Moriyama B., Kontoyiannis D.P. (2016). Candida arthritis: analysis of 112 pediatric and adult cases. Open Forum Infect Dis.

[19] Gamaletsou M.N., Kontoyiannis D.P., Sipsas N.V., Moriyama B., Alexander E., Roilides E. (2012). Candida osteomyelitis: analysis of 207 pediatric and adult cases (1970-2011). Clin Infect Dis.

[20] Zhang C., Lin Y., Huang C., Huang Z., Fang X., Bai G. (2022). Metagenomic next-generation sequencing assists the diagnosis treatment of fungal osteoarticular infections. Front Cell Infect Microbiol.

[21] Chotanaphuti T., Courtney P.M., Fram B., In den Kleef N.J., Kim T.K., Kuo F.C. (2019). Hip and knee section, treatment, algorithm: proceedings of international consensus on orthopedic infections. J Arthroplast.

[22] Chalupova J., Raus M., Sedlarova M., Sebela M. (2014). Identification of fungal microorganisms by MALDI-TOF mass spectrometry. Biotechnol Adv.

[23] Barker K.R., Kus J.V., Normand A.C., Gharabaghi F., McTaggart L., Rotstein C. (2022). A practical workflow for the identification of Aspergillus, Fusarium, Mucorales by MALDI-TOF MS: database, medium, and incubation optimization. J Clin Microbiol.

[24] Clinical and Laboratory Standards Institute (2017).

[25] Clinical and Laboratory Standards Institute (2018).

[26] Clinical and Laboratory Standards Institute (2020).

[27] Clinical and Laboratory Standards Institute (2008).

[28] Gamaletsou M.N., Rammaert B., Bueno M.A., Moriyama B., Sipsas N.V., Kontoyiannis D.P. (2014). Aspergillus osteomyelitis: epidemiology, clinical manifestations, management, and outcome. J Infect.

[29] Koehler P., Tacke D., Cornely O.A. (2016). Bone and joint infections by Mucorales, Scedosporium, Fusarium and even rarer fungi. Crit Rev Microbiol.

[30] Mishra A., Juneja D. (2023). Fungal arthritis: a challenging clinical entity. World J Orthoped.

[31] Xiao C., Qiao D., Xiong L., Tian W., Wang D., Deng S. (2022). Clinical and microbiological characteristics of Aspergillosis at a Chinese tertiary teaching hospital. Infect Drug Resist.

[32] Rychert J., Slechta E.S., Barker A.P., Miranda E., Babady N.E., Tang Y.W. (2018). Multicenter evaluation of the Vitek MS v3.0 system for the identification of Filamentous fungi. J Clin Microbiol.

[33] Blez D., Bronnimann D., Rammaert B., Zeller V., Delhaes L., Hustache L. (2023). Invasive bone and joint infections from the French Scedosporiosis/lomentosporiosis Observational Study (SOS) cohort: no mortality with long-term antifungal treatment and surgery. Med Mycol.

[34] Pellon A., Ramirez-Garcia A., Buldain I., Antoran A., Martin-Souto L., Rementeria A. (2018). Pathobiology of Lomentospora prolificans: could this species serve as a model of primary antifungal resistance?. Int J Antimicrob Agents.

[35] Malloch D.S.I. (1984). A new species of Scedosporium associated with osteomyelitis in humans. Mycotaxon.

[36] Kakuno S., Imoto W., Teranishi Y., Kakeya H. (2023). Lomentospora prolificans-induced Invasive Fungal Sinusitis. Intern Med.

[37] Johnston N., Rockliff B., Duguid R., Palasanthiran P., Bartlett A.W., Willams P.C. (2025). Successful management of Lomentospora prolificans septic arthritis and osteomyelitis in an immunocompetent child: a case report. Med Mycol Case Rep.

[38] Johnson L.B., Bradley S.F., Kauffman C.A. (1998). Fungaemia due to Cryptococcus laurentii and a review of non-neoformans cryptococcaemia. Mycoses.

[39] Kordossis T., Avlami A., Velegraki A., Stefanou I., Georgakopoulos G., Papalambrou C. (1998). First report of Cryptococcus laurentii meningitis and a fatal case of Cryptococcus albidus cryptococcaemia in AIDS patients. Med Mycol.

[40] Averbuch D., Boekhoutt T., Falk R., Engelhard D., Shapiro M., Block C. (2002). Fungemia in a cancer patient caused by fluconazole-resistant Cryptococcus laurentii. Med Mycol.

[41] Bernal-Martinez L., Gomez-Lopez A., Castelli M.V., Mesa-Arango A.C., Zaragoza O., Rodriguez-Tudela J.L. (2010). Susceptibility profile of clinical isolates of non-Cryptococcus neoformans/non-Cryptococcus gattii Cryptococcus species and literature review. Med Mycol.

[42] Akay B.N., Demirdag H.G., Evren E., Karahan Z.C., Okcu Heper A., Kundakci N. (2019). Deep fungal infection caused by Trichophyton rubrum after heart transplantation: a case report with dermoscopy. Australas J Dermatol.

[43] Azzam K., Parvizi J., Jungkind D., Hanssen A., Fehring T., Springer B. (2009). Microbiological, clinical, and surgical features of fungal prosthetic joint infections: a multi-institutional experience. J Bone Joint Surg Am.

[44] Cho S., Lee H., Hwang J.Y., Choi J.S., Kim H.J., Kim T.W. (2021). Prevalence and characteristics of onychomycosis in patients with knee osteoarthritis: a single-centre prospective cross-sectional study. Acta Derm Venereol.

[45] Tsantes A.G., Papadopoulos D.V., Markou E., Zarokostas K., Sokou R., Trikoupis I. (2022). Aspergillus spp. osteoarticular infections: an updated systematic review on the diagnosis, treatment and outcomes of 186 confirmed cases. Med Mycol.

[46] Teixeira M.M., Carvalho D.T., Sousa E., Pinto E. (2022). New antifungal agents with azole moieties. Pharmaceuticals.

[47] Chapman S.W., Dismukes W.E., Proia L.A., Bradsher R.W., Pappas P.G., Threlkeld M.G. (2008). Infectious diseases society of A. clinical practice guidelines for the management of blastomycosis: 2008 update by the infectious Diseases Society of America. Clin Infect Dis.

[48] Hassan M.M., Harrington N.E., Sweeney E., Harrison F. (2020). Predicting antibiotic-associated virulence of Pseudomonas aeruginosa using an ex vivo lung biofilm model. Front Microbiol.

[49] Sienkiewicz B.M., Lapinski L., Wiela-Hojenska A. (2016). Comparison of clinical pharmacology of voriconazole and posaconazole. Contemp Oncol.

[50] Zhang S., Zhang L., Yusufu A., Hasimu H., Wang X., Abliz P. (2024). Clinical distribution and drug susceptibility characterization of invasive Candida isolates in a tertiary hospital of Xinjiang province. Infect Drug Resist.

[51] Olender A., Bogut A., Dabrowski W., Pietrzak D.J., Szukala M., Wojtowicz-Bobin M. (2025). Analysis of antifungal drug resistance among Candida spp. and other pathogenic yeasts isolated from patients in Eastern Poland: diagnostic problems. Infect Drug Resist.

[52] Clark N.M., Grim S.A., Lynch J.P. (2015). Posaconazole: use in the prophylaxis and treatment of fungal infections. Semin Respir Crit Care Med.

[53] Firinu D., Massidda O., Lorrai M.M., Serusi L., Peralta M., Barca M.P. (2011). Successful treatment of chronic mucocutaneous candidiasis caused by azole-resistant Candida albicans with posaconazole. Clin Dev Immunol.

[54] Arranz-Caso J.A., Lopez-Pizarro V.M., Gomez-Herruz P., Garcia-Altozano J., Martinez-Martinez J. (1996). Candida albicans osteomyelitis of the zygomatic bone. A distinctive case with a possible peculiar mechanism of infection and therapeutic failure with fluconazole. Diagn Microbiol Infect Dis.

[55] Kim Y.M., Guk T., Jang M.K., Park S.C., Lee J.R. (2024). Targeted delivery of amphotericin B-loaded PLGA micelles displaying lipopeptides to drug-resistant Candida-infected skin. Int J Biol Macromol.

[56] Avunduk A.M., Beuerman R.W., Warnel E.D., Kaufman H.E., Greer D. (2003). Comparison of efficacy of topical and oral fluconazole treatment in experimental Aspergillus keratitis. Curr Eye Res.

