# Supplementary material for: Species distribution, antifungal susceptibility, and clinical profiles of patients with osteoarticular fungal Infections: A retrospective study
Source: New Microbes New Infect. 2025 Nov 21;68:101676. doi: 10.1016/j.nmni.2025.101676 (PMC12686614; doi:10.1016/j.nmni.2025.101676)
Supplement: Multimedia component 1 [file mmc1.docx]

**STROBE Statement**—checklist of items that should be included in reports of observational studies

|  | Item No. | Recommendation | Page  No. | Relevant text from manuscript |
| --- | --- | --- | --- | --- |
| **Title and abstract** | 1 | (*a*) Indicate the study’s design with a commonly used term in the title or the abstract | 1 | Species Distribution, Antifungal Susceptibility, and Clinical Profiles of Patients with Osteoarticular Fungal Infections: A Retrospective Study |
|  |  | (*b*) Provide in the abstract an informative and balanced summary of what was done and what was found | 1-2 | **Abstract**  Background: Osteoarticular fungal infections (OAFIs), including fungal osteomyelitis and septic arthritis, represent uncommon but clinically significant complications in musculoskeletal care. Current management remains challenging due to limited evidence guiding antifungal selection. This study aims to characterize the epidemiological patterns, clinical features, and antifungal susceptibility profiles of OAFIs, with particular focus on the critical role of in vitro susceptibility testing in determining treatment outcomes.  Methods: A retrospective study of patients was conducted with OAFIs treated between January 2020 and February 2024, analyzing clinical manifestations, surgical interventions, and associated risk factors. Fungal identification was performed using matrix-assisted laser desorption/ionization time-of-flight mass spectrometry (MALDI-TOF MS), followed by broth microdilution antifungal susceptibility testing for amphotericin B (AMB), fluconazole (FLC), voriconazole (VRC), and posaconazole (POS). Minimum inhibitory concentration (MIC) values were interpreted according to CLSI guidelines to determine susceptibility profiles and identify potential resistance mechanisms.  Results: Sixty fungal isolates were isolated from 60 patients with OAFIs including Candida spp. (n=40, 66.7%), Aspergillus spp. (n=14, 23.3%), Cryptococcus neoformans (n=2, 3.3%), Trichophyton rubrum (n=2, 3.3%), Lomentospora prolificans (n=1, 1.7%), and Cryptococcus laurentii (n=1, 1.7%). The isolates were obtained from joint fluid (n=48, 80%) and inflammatory lesions (n=12, 20%). Antifungal susceptibility testing demonstrated highest MIC values for FLC but susceptibility profiles for VRC and POS against all fungal isolates. Statistical analysis revealed significant differences in VRC and POS activity among Candida, Aspergillus, and Cryptococcus spp. (F = 15.78, P<0.01; F = 66.88, P<0.0001). VRC activity did not differ between Candida and Aspergillus spp., but both were lower than against Cryptococcus spp. (P<0.05 and P<0.05). POS activity was higher against Candida than Aspergillus (P<0.001) and Cryptococcus spp. (P<0.0001), and higher against Cryptococcus than Aspergillus (P<0.05). Systemic comorbidities were common (73.3%), one patient was HIV-positive, and three had only localized superficial fungal infections.  Conclusion: We concluded that Candida albicans and Aspergillus fumigatus as the predominant pathogens in OAFIs, while rare species including Cryptococcus neoformans, Lomentospora prolificans, and Cryptococcus laurentii were also isolated from OAFI cases. Antifungal susceptibility testing revealed VRC and POS as potentially effective therapeutic options for OAFIs. These findings underscore the need for early detection of rare fungal pathogens, susceptibility-guided therapy, and continuous resistance surveillance in managing OAFIs in non-immunodeficient patients. |
| Introduction | | | |  |
| Background/rationale | 2 | Explain the scientific background and rationale for the investigation being reported | 3-4 | “Osteoarticular fungal infections (OAFIs)... are rare but serious infections that may lead to substantial disability... Diagnosing and treating OAFIs present considerable challenges... due to the often subtle and gradual onset, leading to delayed recognition... a consistent rise in reported cases has been observed over the past ten years... Current literature on OAFIs is limited to small case series and individual case reports, resulting in lower-quality evidence.” |
| Objectives | 3 | State specific objectives, including any prespecified hypotheses | 4 | “This study aimed to characterize the epidemiological patterns, clinical manifestations, and antifungal susceptibility profiles of OAFIs, with particular emphasis on the role of *in vitro* susceptibility testing in guiding therapeutic decision. Furthermore, through comprehensive retrospective analysis, we aimed to establish evidence-based recommendations to assist clinicians in optimizing antifungal treatment regimens.” |
| Methods | | | |  |
| Study design | 4 | Present key elements of study design early in the paper | 5 | “All retrospective analyses were conducted... the study included all cases of OAFIs diagnosed and treated at our institution between January 2020 and February 2024.” |
| Setting | 5 | Describe the setting, locations, and relevant dates, including periods of recruitment, exposure, follow-up, and data collection | 5 | **Setting/Location:** “Shanxi Provincial People’s Hospital.” **Dates:** “included all cases... between January 2020 and February 2024.” **Follow-up:** “Patients whose follow-up period was less than one month... were excluded.” |
| Participants | 6 | (*a*) *Cohort study*—Give the eligibility criteria, and the sources and methods of selection of participants. Describe methods of follow-up  *Case-control study*—Give the eligibility criteria, and the sources and methods of case ascertainment and control selection. Give the rationale for the choice of cases and controls  *Cross-sectional study*—Give the eligibility criteria, and the sources and methods of selection of participants | 5-6 | **Eligibility Criteria:** “OAFIs was defined based on the criteria outlined by Gamaletsou et al., including (1) relevant clinical presentation, (2) corresponding radiographic findings, and (3) the presence of fungi has been confirmed by culturing bone tissue or metal hardware samples...” **Sources/Methods of Selection:** “the study included all cases of OAFIs diagnosed and treated at our institution between January 2020 and February 2024.” **Exclusion Criteria:** “Patients whose follow-up period was less than one month following the initial surgery were excluded from the study.” |
|  |  | (*b*) *Cohort study*—For matched studies, give matching criteria and number of exposed and unexposed  *Case-control study*—For matched studies, give matching criteria and the number of controls per case | / | Our study, as described, does not involve matching, so this item is not applicable. |
| Variables | 7 | Clearly define all outcomes, exposures, predictors, potential confounders, and effect modifiers. Give diagnostic criteria, if applicable | 10 | Outcomes: “Treatment success was defined by the following criteria: (1) absence of infection-related symptoms... (2) normalization of inflammatory markers... (3) no radiological evidence of infection...” “Treatment failure was defined as infection relapse, the need for additional surgical intervention...”  Exposures/Predictors: Fungal species, antifungal agents (AMB, FLC, VRC, POS), type of infection (PJI, PFOI, SFOI).  Diagnostic Criteria: Provided for OAFIs and PJI, referencing Gamaletsou et al. and MSIS guidelines. |
| Data sources/ measurement | 8* | For each variable of interest, give sources of data and details of methods of assessment (measurement). Describe comparability of assessment methods if there is more than one group | 7-9 | Data Sources: “anonymized clinical records.” Methods of Assessment: Demographics/Clinical Data: Collected from records. Fungal Identification: “Species identification was confirmed by MALDI-TOF mass spectrometry...” Antifungal Susceptibility: “broth microdilution techniques according to Clinical and Laboratory Standards Institute guidelines (CLSI), M27-A3, S4 and M38-A2, M59.” |
| Bias | 9 | Describe any efforts to address potential sources of bias | 7-9 | The study mitigated selection bias by including “all cases of OAFIs diagnosed and treated at our institution” within the specified timeframe (a consecutive series). Measurement bias was addressed by using standardized, reference methods (CLSI, MALDI-TOF) for laboratory analyses. |
| Study size | 10 | Explain how the study size was arrived at | 5 | The sample size was not predetermined. It included all eligible cases presenting during the study period: “the study included all cases of OAFIs diagnosed and treated at our institution between January 2020 and February 2024.” |

Continued on next page

| Quantitative variables | 11 | Explain how quantitative variables were handled in the analyses. If applicable, describe which groupings were chosen and why | 9-11 | MIC values were measured and interpreted using CLSI criteria and ECVs. For statistical tests, quantitative data are described as “means ± standard deviations (SD)”. Normality and variance homogeneity were assessed to choose between ANOVA and Kruskal-Wallis tests. |
| --- | --- | --- | --- | --- |
| Statistical methods | 12 | (*a*) Describe all statistical methods, including those used to control for confounding | 10-11 | “Statistical analysis was performed using SPSS software (version 18.0)... one-way analysis of variance (ANOVA) was applied... otherwise, the Kruskal–Wallis test was used... Post hoc pairwise comparisons were conducted with Bonferroni correction... Student’s t-test... A two-tailed *P* < 0.05 was considered statistically significant.” |
|  |  | (*b*) Describe any methods used to examine subgroups and interactions | 10-11 | Post-hoc pairwise comparisons with Bonferroni correction were used following ANOVA/Kruskal-Wallis tests to examine differences between specific fungal species subgroups (e.g., *Candida*, *Aspergillus*, *Cryptococcus*). |
|  |  | (*c*) Explain how missing data were addressed | / | Not Applicable. |
|  |  | (*d*) *Cohort study*—If applicable, explain how loss to follow-up was addressed  *Case-control study*—If applicable, explain how matching of cases and controls was addressed  *Cross-sectional study*—If applicable, describe analytical methods taking account of sampling strategy | 5-6 | Loss to follow-up was addressed by excluding “Patients whose follow-up period was less than one month" and those “lost to follow-up” |
|  |  | (*e*) Describe any sensitivity analyses | 9-11 | “Essential agreement (EA) was defined as discrepancies in MIC results of no more than ±2-fold dilutions between two methods……For the remaining strains, no established drug-specific breakpoints were available; therefore, only MIC values were reported.” And “Statistical analysis was performed using SPSS software (version 18.0; SPSS Inc., Chicago, IL, USA)……A two-tailed *P* < 0.05 was considered statistically significant.” |
| Results | | | | |
| Participants | 13* | (a) Report numbers of individuals at each stage of study—eg numbers potentially eligible, examined for eligibility, confirmed eligible, included in the study, completing follow-up, and analysed | 11 | “A total of 65 patients were identified from the registry. Four were excluded due to a follow-up period of less than one month, one was lost to follow-up, leaving 60 patients for analysis.” |
|  |  | (b) Give reasons for non-participation at each stage | 11 | “Four were excluded due to a follow-up period of less than one month, one was lost to follow-up.” |
|  |  | (c) Consider use of a flow diagram | 11 | The study flowchart is shown in Figure 1. |
| Descriptive data | 14* | (a) Give characteristics of study participants (eg demographic, clinical, social) and information on exposures and potential confounders | 11-12 | Demographics: “The male-to-female ratio was 29 (48%) to 31 (52%). According to the age... 3 (5%)... less than 40 years old, 26 (43%)... between 40-60 years old, and 31 (52%)... over 60 years old.”  Clinical Characteristics/Exposures: “29 had periprosthetic joint infection (PJI)... 23 had primary fungal osteoarticular infection (PFOI)... 8 had secondary fungal osteoarticular infection (SFOI).” Details of prior surgeries are provided.  Potential Confounders (Comorbidities): “47 (78.3%) had at least one comorbid condition... including coronary heart disease (CHD), hypertension (HT), diabetes mellitus (DM)... HIV infection (1 patient)...” Inflammatory marker levels (CRP, ESR, synovial WBC) are also reported. |
|  |  | (b) Indicate number of participants with missing data for each variable of interest | / | Not explicitly mentioned in the provided text. The results assume complete data for the reported variables. |
|  |  | (c) *Cohort study*—Summarise follow-up time (eg, average and total amount) | 15 | Over a median follow-up of 41.4 months (range: 19–62 months). |
| Outcome data | 15* | *Cohort study*—Report numbers of outcome events or summary measures over time | 14-15 | The overall treatment success rate was 95% (54/60)... Treatment failure was documented in two patients... One patient... died... three patients... were managed successfully with spacer retention. |
|  |  | *Case-control study—*Report numbers in each exposure category, or summary measures of exposure |  |  |
|  |  | *Cross-sectional study—*Report numbers of outcome events or summary measures |  |  |
| Main results | 16 | (*a*) Give unadjusted estimates and, if applicable, confounder-adjusted estimates and their precision (eg, 95% confidence interval). Make clear which confounders were adjusted for and why they were included | 15 | The results presented are primarily descriptive (e.g., species distribution, MIC values, success rates). The text reports statistical comparisons of MIC values (“F = 15.78, *P*<0.01; F = 66.88, *P*<0.0001”) but these are unadjusted estimates from ANOVA. The manuscript does not report confounder-adjusted estimates (e.g., from multivariate regression) for the association between exposures and the outcome of treatment success/failure. |
|  |  | (*b*) Report category boundaries when continuous variables were categorized | 11 | Age was categorized: “less than 40 years old,” “between 40-60 years old,” and “over 60 years old.” |
|  |  | (*c*) If relevant, consider translating estimates of relative risk into absolute risk for a meaningful time period | / | Not applicable, as the study does not present estimates of relative risk. The primary outcomes are reported as descriptive proportions (e.g., treatment success rate) and MIC distributions. |

Continued on next page

| Other analyses | 17 | Report other analyses done—eg analyses of subgroups and interactions, and sensitivity analyses | 13-15 | Subgroup Analyses: Statistical comparisons of antifungal activity (MIC values) were performed between major fungal subgroups. “Statistical analysis revealed significant differences in VRC and POS activity among Candida, Aspergillus, and Cryptococcus spp. (F = 15.78, *P*<0.01; F = 66.88, *P*<0.0001). VRC activity did not differ between *Candida* and *Aspergillus* spp., but both were lower than against *Cryptococcus* spp. (*P*<0.05 and P<0.05). POS activity was higher against *Candida* than *Aspergillus* (*P*<0.001) and *Cryptococcus* spp. (*P*<0.0001), and higher against *Cryptococcus* than *Aspergillus* (*P*<0.05).”  Other Analyses: The antifungal susceptibility profiles (MIC_50_, MIC_90_, GM) were reported and compared for subgroups of individual species, including *Candida albicans*, *Candida parapsilosis*, *Candida tropicalis*, *Aspergillus fumigatus*, and *Aspergillus flavus*. |
| --- | --- | --- | --- | --- |
| Discussion | | | | |
| Key results | 18 | Summarise key results with reference to study objectives | 15-16 | This study systematically characterized the distribution and antifungal susceptibility of osteoarticular fungal infections (OAFIs), a rare but clinically significant condition associated with diagnostic challenges and high rates of misdiagnosis. We identified 40 *Candida* isolates, with *C. albicans*, *C. tropicalis*, and *C. flavus* being predominant, and 14 *Aspergillus* isolates as the main filamentous fungi. Emerging pathogens, such as *Lomentospora prolificans*, *Cryptococcus laurentii*, and *Trichophyton rubrum*, were also isolated, underscoring the expanding spectrum of OAFIs. Antifungal susceptibility testing revealed that 73.3% of pathogens were sensitive to voriconazole (VRC), yet discrepancies between in vitro susceptibility and clinical outcomes were noted, particularly in cases where posaconazole (POS) achieved therapeutic success following VRC failure. The majority of patients (78.3%) had systemic comorbidities, indicating that comorbidity is a major risk factor for OAFIs. Collectively, these findings contribute to refining the epidemiological and therapeutic landscape of OAFIs, while highlighting the complexity of treatment decision-making. |
| Limitations | 19 | Discuss limitations of the study, taking into account sources of potential bias or imprecision. Discuss both direction and magnitude of any potential bias | 22 | Several limitations must be acknowledged. First, fungal identification was primarily conducted using MALDI-TOF MS without additional confirmation of *Candida* species by CHROMagar™ *Candida*, potentially limiting taxonomic resolution. Second, antifungal susceptibility testing excluded echinocandins, restricting comprehensive evaluation of resistance patterns. Third, as a retrospective study with a modest sample size (n = 60), the statistical power was limited, and some associations may not have reached significance. Furthermore, selection bias cannot be ruled out, as cases were drawn from a single tertiary care center, which may limit representativeness. These factors may influence both the direction and magnitude of observed associations, particularly regarding generalization of antifungal resistance profiles. |
| Interpretation | 20 | Give a cautious overall interpretation of results considering objectives, limitations, multiplicity of analyses, results from similar studies, and other relevant evidence | 23-24 | Taken together, our findings suggest that OAFIs are not only associated with common pathogens such as *Candida* and *Aspergillus* species but also with rare and emerging fungi, necessitating heightened clinical awareness. The discordance between in vitro susceptibility and clinical response highlights the limitations of current antifungal testing methodologies and emphasizes the importance of integrating pharmacokinetic, host, and pathogen-specific factors into therapeutic decisions. While voriconazole and amphotericin B remain cornerstones of treatment, posaconazole showed clinical benefit in cases where first-line azoles failed, indicating a potential underrecognized role in OAFIs. These results are consistent with reports from other studies that highlight the variability in treatment response and the importance of individualized therapy. Nevertheless, the retrospective nature, lack of echinocandin data, and limited sample size underscore the need for prospective, multicenter studies and meta-analyses to validate these observations. |
| Generalisability | 21 | Discuss the generalisability (external validity) of the study results | 23-24 | Given the rarity of OAFIs and the single-center design, caution is warranted in extrapolating our findings to broader populations. The pathogen distribution and antifungal susceptibility patterns observed in this Chinese cohort may not fully reflect epidemiological or therapeutic profiles in other geographic regions, where environmental, genetic, and healthcare factors differ. However, the identification of emerging pathogens, the high prevalence of comorbidities, and the observed discrepancies between laboratory and clinical outcomes provide insights of relevance to clinicians worldwide managing OAFIs. Our study thus contributes valuable, albeit preliminary, evidence that can inform both regional and international clinical practice, pending confirmation by larger multicenter studies. |
| Other information | |  | | |
| Funding | 22 | Give the source of funding and the role of the funders for the present study and, if applicable, for the original study on which the present article is based | 29 | This work was supported by the Basic Research Program of Shanxi Province (No. 202403021212208). |

*Give information separately for cases and controls in case-control studies and, if applicable, for exposed and unexposed groups in cohort and cross-sectional studies.

**Note:** An Explanation and Elaboration article discusses each checklist item and gives methodological background and published examples of transparent reporting. The STROBE checklist is best used in conjunction with this article (freely available on the Web sites of PLoS Medicine at http://www.plosmedicine.org/, Annals of Internal Medicine at http://www.annals.org/, and Epidemiology at http://www.epidem.com/). Information on the STROBE Initiative is available at www.strobe-statement.org.
