# Supplementary material for: Species distribution, antifungal susceptibility, and clinical profiles of patients with osteoarticular fungal Infections: A retrospective study
Source: New Microbes New Infect. 2025 Nov 21;68:101676. doi: 10.1016/j.nmni.2025.101676 (PMC12686614; doi:10.1016/j.nmni.2025.101676)
Supplement: Multimedia component 2 [file mmc2.doc]

**Supplemental Table 1**. Demographic and clinical data of the 60 patients with fungal osteoarticular infection (FOI).

| **No.** | **Sex/Age** | **Type** | **Primary diagnosis** | **Underlying condition** | **Primary surgery** | **Extra surgery/antibioticx** | **Fungal species** | **Other pathogens** | **SF WBC (x106/L)** | **PMN (%)** | **CRP (mg/L)** | **ESR (mm/h)** | **Treatment** | **FU**  **(months)** | **Outcome** |
| --- | --- | --- | --- | --- | --- | --- | --- | --- | --- | --- | --- | --- | --- | --- | --- |
| 1 | F/61 | PJI | TB | HT | TKA(R) | Multiple Debridement | *Candida albicans* | / | 5267 | 45.3 | 23.5 | 73 | TR+POS | 43 | Success |
| 2 | F/58 | PJI | OA | HT | TKA(L) | / | *Candida albicans* | / | 7593 | 67.9 | 18.7 | 24 | TR+FLC | 41 | Success |
| 3 | M/62 | PJI | OA | HT | TKA(L) | / | *Candida albicans* | / | 4321 | 52.4 | 34.2 | 105 | TR+POS | 57 | Success |
| 4 | F/77 | PJI | OA | DM | UKA(L) | / | *Candida parapsilosis* | / | 6854 | 73.1 | 12.9 | 47 | TR+VRC | 55 | Success |
| 5 | F/79 | PJI | OA | DM | THA(L) | 1 DAIR + Debridement + spacer. Long-term cefazolin. | *Candida tropicalis* | *Staphlycoccus cohnii* | 9032 | 61.6 | 39.6 | 89 | TR+VRC | 40 | Unsuccess |
| 6 | F/35 | PJI | TB | DM | THA(L) | PFNA removal + spacer + debridement. Long-term cefazolin | *Aspergillus fumigatus* | *Staphlycoccus aureus* | 8125 | 48.8 | 27.3 | 12 | OR+VRC | 29 | Spacer retention |
| 7 | M/67 | PFOI | ONFH | Hyperthyroidism | THA(L) | Screw removal + spacer + debridement. Long-term ampicillin | *Candida tropicalis* | *Enterococcus faecalis* | 4789 | 55.5 | 15.8 | 61 | TR+VRC | 27 | Spacer retention |
| 8 | M/45 | PJI | RA | HT, chronic pneumonia, liver cyst | TKA(R) | 1 DAIR + spacer +multiple Debridement. Long-term doxycycline | *Aspergillus fumigatus* | *Mycoplasma hominis* | 6472 | 40.2 | 31.4 | 96 | Long-term VRC | 37 | Spacer retention |
| 9 | F/60 | SFOI | ACI, tear | Renal dysfunction | ACLR(R) | 1 Debridement + 1 DAIR | *Aspergillus flavus* | / | 5801 | 79.4 | 20.1 | 33 | TR+VRC | 56 | Success |
| 10 | F/81 | PFOI | Chronic hip arthiritis | DM, hypoalbuminemia | THA(L) | Multiple debridement | *Aspergillus fumigatus* | */* | 7346 | 42.7 | 36.9 | 78 | Arthroscopic debridement+VRC | 44 | Success |
| 11 | F/82 | PJI | OA | HT, DM | TKA(R) | / | *Cryptococcus laurentii* | */* | 4500 | 66.3 | 11.3 | 52 | Debridement+fusion+VRC | 58 | Success |
| 12 | M/37 | PJI | TB | DM | TKA(R) | / | *Aspergillus fumigatus* | / | 4925 | 54.9 | 28.6 | 117 | Long-term VRC. Refused surgery | 30 | Success |
| 13 | M/71 | PFOI | ONFH | pneumonia | None | / | *Candida albicans* | / | 8773 | 72.8 | 16.4 | 7 | VRC. Refused surgery | 31 | Success |
| 14 | M/45 | PFOI | RA | DM, bilateral kidney stone, HBV | Debridement | / | *Candida albicans* | / | 9501 | 41.5 | 33.7 | 84 | TR+POS. Refused reimplantation | 42 | Success |
| 15 | M/58 | PJI | Ankle TB | HIV | Bilateral TKA | / | *Cryptococcus neoformans* | / | 6148 | 69.2 | 22.9 | 39 | Arthroscopic debridement+VRC | 52 | Success |
| 16 | M/60 | SFOI | Rib malignnat neoplasm | None | PFNA (L) | / | *Candida parapsilosis* | / | 5390 | 78.6 | 38.5 | 101 | PFNA+ FLC. | 38 | Success |
| 17 | M/65 | PFOI | Rib TB | Uremia, DM, HT, hypoalbuminemia, anemia | None | / | *Candida parapsilosis* | / | 3100 | 47.1 | 14.2 | 16 | TR+VRC. Refused reimplantation. | 26 | Success |
| 18 | F/51 | PJI | Intertrochanteric fracture | None | TKA (L) | 1 DAIR | *Candida parapsilosis* | / | 2560 | 60 | 29.8 | 67 | TKA+VRC. | 25 | Success |
| 19 | M/47 | PFOI | Femoral neck fracture | Tinea pedis | THA (L) | 1 DAIR + debridement + spacer. Long-term cefazolin | *Candida tropicalis* | *Staphlycoccus cohnii* | 11062 | 50.4 | 19.6 | 93 | TKA+POS | 37 | Success |
| 20 | F/80 | PFOI | ONFH | HT, DM | Debridement | PFNA removal + spacer + debridement. Long-term cefazolin | *Candida parapsilosis* | *Staphlycoccus aureus* | 8826 | 76.5 | 35.1 | 28 | TR+VRC | 49 | Success |
| 21 | F/87 | SFOI | Osteomyelitis | HT, DM | THA(L) | / | *Candida tropicalis* | / | 4210 | 63.7 | 13.7 | 55 | TR+VRC | 43 | Dead (acute myocardial infarction) |
| 22 | F/65 | PJI | OA | HT, DM | TKA(R) | / | *Aspergillus fumigatus* | */* | 7985 | 44 | 25.4 | 112 | TR+VRC | 31 | Success |
| 23 | M/63 | PFOI | OA | HBV, DM | None | / | *Aspergillus flavus* | / | 6837 | 57.8 | 32.8 | 41 | TR+VRC | 56 | Success |
| 24 | M/52 | PJI | Chronic hip arthiritis | HT | TKA(L) | / | *Aspergillus flavus* | / | 5558 | 70.2 | 17.9 | 79 | TR+VRC | 28 | Success |
| 25 | M/51 | PJI | Septic knee arthritis | Tinea pedis  (*Candida albicans*) | TKA(L) | / | *Candida tropicalis* | / | 7341 | 49.9 | 37.3 | 19 | TKA+VRC | 37 | Success |
| 26 | F/62 | PFOI | OA | HT | TKA(L) | / | *Candida albicans* | / | 4102 | 75.1 | 21.6 | 86 | Arthroscopic debridement+POS | 44 | Success |
| 27 | M/55 | PFOI | TB | HT | TKA(L) | / | *Candida albicans* | / | 9204 | 64.8 | 10.8 | 58 | THA + VRC | 54 | Success |
| 28 | F/70 | PJI | ONFH | DM | UKA(L) | / | *Candida albicans* | / | 6592 | 53.6 | 30.2 | 110 | TR+POS | 39 | Success |
| 29 | M/81 | PJI | Fracture | DM, Tinea corporis | TKA(L) | / | *Candida albicans* | / | 7,272 | 80 | 8.2 | 3 | TR+VRC | 28 | Success |
| 30 | F/79 | PFOI | OA | CHD, Renal transplantation | Debridement | / | *Cryptococcus neoformans* | / | 5094 | 46.2 | 15.6 | 15 | TR+VRC | 21 | Success |
| 31 | F/71 | PJI | Fracture | HT | UKA(L) | 1 DAIR | *Candida albicans* | / | 15070 | 68.4 | 22.3 | 7 | TR+VRC | 42 | Success |
| 32 | F/68 | SFOI | OA | Renal dysfunction | Debridement | / | *Lomentospora prolificans* | / | 8875 | 58.1 | 6.5 | 18 | TR+VRC | 31 | Success |
| 33 | M/67 | PFOI | OA | Renal dysfunction | None | / | *Candida albicans* | / | 3660 | 74.3 | 27.1 | 1 | TR+VRC | 19 | Success |
| 34 | F/66 | PJI | Fracture | HT | TKA (L) | / | *Aspergillus flavus* | / | 8352 | 41.7 | 12.8 | 12 | TR+VRC | 58 | Success |
| 35 | M/65 | PJI | OA | None | TKA(L) | Multiple debridement | *Candida parapsilosis* | / | 7754 | 62.5 | 19.4 | 9 | TR+VRC | 40 | Unsuccess |
| 36 | M/65 | PFOI | OA | None | None | / | *Candida albicans* | / | 3841 | 77.2 | 10.7 | 0 | TR+POS | 39 | Success |
| 37 | M/65 | SFOI | Intertrochanteric fracture | HT | PFNA (L) | / | *Aspergillus fumigatus* | / | 4872 | 52.1 | 25.9 | 5 | TR+VRC | 33 | Success |
| 38 | F/64 | PJI | OA | Hyperthyroidism | TKA(R) | Multiple debridement | *Candida tropicalis* | / | 5681 | 43.9 | 16.2 | 16 | TKA+VRC | 32 | Success |
| 39 | M/64 | SFOI | OA | HT | TKA |  | *Aspergillus fumigatus* | / | 4921 | 65.6 | 9.1 | 10 | TR+POS | 28 | Success |
| 40 | F/62 | PFOI | OA | None | None | Debridement, antibiotics | *Candida albicans* | / | 8370 | 71.4 | 14.4 | 4 | TR+VRC | 38 | Success |
| 41 | F/62 | SFOI | Fracture | Tinea pedis  (*Trichophyton rubrum*) | TKA (L) | / | *Trichophyton rubrum* | */* | 7356 | 59.3 | 23.5 | 17 | TR+VRC | 46 | Success |
| 42 | M/61 | SFOI | Intertrochanteric fracture | HT | TKA (R) | / | *Candida albicans* | / | 6021 | 40.9 | 7.3 | 8 | TR+VRC | 27 | Success |
| 43 | M/61 | PFOI | Fracture | HT | TKA(L) | / | *Candida albicans* | / | 8423 | 48.3 | 28 | 19 | TR+POS | 38 | Success |
| 44 | F/59 | PFOI | OA | HT | None | Debridement, antibiotics | *Candida parapsilosis* | / | 9187 | 72 | 11.6 | 2 | TR+VRC | 41 | Success |
| 45 | M/58 | PFOI | Femoral neck fracture | HT | None | / | *Candida albicans* | / | 4563 | 66.7 | 18.2 | 14 | TR+VRC | 62 | Success |
| 46 | F/55 | PFOI | Fracture | HT | None | Debridement, antibiotics | *Candida albicans* | / | 6732 | 55.2 | 24.7 | 11 | TR+ POS | 58 | Success |
| 47 | M/54 | PFOI | OA | None | None | Debridement, antibiotics | *Candida albicans* | / | 8501 | 42.4 | 5.9 | 6 | TR+POS | 47 | Success |
| 48 | F/52 | PJI | OA | None | TKA(L) | / | *Candida albicans* | / | 5148 | 67.2 | 29.8 | 13 | TR+POS | 41 | Success |
| 49 | F/45 | PJI | OA | HT | TKA(L) | / | *Candida albicans* | / | 7894 | 79.1 | 13.5 | 20 | TR+VRC | 54 | Success |
| 50 | F/86 | PFOI | OA | None | None | / | *Candida albicans* | / | 9325 | 50.6 | 20.6 | 3 | TR+POS | 42 | Success |
| 51 | M/54 | PJI | OA | None | TKA(R) | Multiple Debridement | *Candida albicans* | / | 4785 | 74.6 | 26.4 | 8 | TR+POS | 41 | Success |
| 52 | F/53 | PFOI | OA | CHD | None | / | *Aspergillus fumigatus* | */* | 6150 | 61.2 | 17.1 | 15 | TR+VRC | 49 | Success |
| 53 | F/53 | PJI | Femoral neck fracture | CHD | TKA(L) | / | *Aspergillus flavus* | / | 8290 | 45.7 | 21.9 | 1 | TR+VRC | 52 | Success |
| 54 | M/55 | PJI | Fracture | None | TKA(L) | / | *Candida parapsilosis* | / | 5732 | 54.3 | 8.7 | 10 | TR+VRC | 40 | Success |
| 55 | M/55 | PFOI | OA | None | None | Debridement, antibiotics | *Candida albicans* | / | 4019 | 78.4 | 14.9 | 17 | TR+AMB | 60 | Success |
| 56 | F/57 | PFOI | OA | None | None | Debridement, antibiotics | *Aspergillus fumigatus* | / | 7624 | 49.5 | 30 | 6 | TR+VRC | 51 | Success |
| 57 | F/57 | PJI | OA | None | TKA(L) | / | *Candida albicans* | / | 6904 | 70.9 | 9.6 | 19 | TR+VRC | 46 | Success |
| 58 | M/58 | PJI | OA | CHD | TKA(R) | / | *Candida albicans* | / | 8295 | 63.1 | 15.3 | 75 | TR+VRC | 37 | Success |
| 59 | F/49 | PJI | RA | DM, tinea cruris (*Trichophyton rubrum*) | TKA(L) | 1 DAIR | *Trichophyton rubrum* | */* | 5406 | 58.5 | 19.8 | 35 | Long-term VRC | 43 | Success |
| 60 | M/77 | PJI | ONFH | HT | TKA(L) | 1 DAIR + Debridement + spacer. Long-term ceftriaxone. | *Candida albicans* | *Klebsiella pneumoniae* | 7512 | 76.9 | 23.7 | 29 | TR+POS | 51 | Success |

HT, hypertension; DM, diabetes; HIV, human immunodeficiency virus; HBV, hepatitis B; OA, osteoarthritis; TB, tuberculosis; RA, rheumatoid arthritis; ONFH, osteonecrosis of the femoral head; TKA, total knee arthroplasty; UKA, unicompartmental knee arthroplasty; THA, total hip arthroplasty; PFNA, proximal femoral nail antirotation; ACLR, anterior cruciate ligament reconstruction; DAIR, debridement, antibiotics, and implant retention; abx, antibiotics; SF, synovial fluid; WBC, white blood cell; PMN, polymorphonuclear leukocyte; FU, follow-up; OR, one-stage revision; TR, two-stage revision; FLC, fluconazole, VRC, voriconazole; AMB, amphotericin B; POS, posaconazole. Spacer retention, antifungal-impregnated cement spacer retention; Long-term abx: therapy exceeding 6 months.
